# ﻿Two new species of the genus *Veraphis* Casey (Coleoptera, Staphylinidae, Scydmaeninae) from Korea

**DOI:** 10.3897/zookeys.1224.142859

**Published:** 2025-01-28

**Authors:** Ui-Joung Byeon, Jong-Seok Park

**Affiliations:** 1 Department of Biological Sciences and Biotechnology, Chungbuk National University, 1 Chungdae-ro, Seowon-gu, Cheongju-si, Chungbuk-do, 28644, Republic of Korea Chungbuk National University Cheongju-si Republic of Korea

**Keywords:** Ant-like stone beetles, distribution map, Eutheiini, morphology, taxonomy

## Abstract

Two new species, *Veraphisodaesanensis***sp. nov.** and *Veraphismyeonggiensis***sp. nov.**, of the ant-like stone beetle, are described from the Korean Peninsula. The Korean fauna of *Veraphis* Casey now comprises three species, including *V.engelmarkikoreanus*. This study provides habitus images and aedeagus illustrations of the new species.

## ﻿Introduction

The genus *Veraphis* Casey, 1897 (Staphylinidae: Scydmaeninae: Eutheiini) is exclusively distributed in the Northern Hemisphere, comprising 35 species (including three subspecies). Eight species occur in the Nearctic region, one species in Scandinavia, and the remaining species are found in Far East Russia, Japan, North Korea, Siberia, Mongolia, and China (Sawada 1962; Franz 1971; Hisamatsu 1985; Kurbatov 1995, 2006; Jałoszyński and Hoshina 2005; Jałoszyński 2009, 2012, 2013, 2014a, 2019, 2024). According to Jałoszyński and Hoshina (2005), the Japanese *Veraphis* species are grouped into three distinct species groups, except for *V.engelmarki* Franz and *V.ishikawai* Hisamatsu. Some species, *Veraphisspinosus* Jałoszyński, *V.qinghaiensis* Jałoszyński, *V.calcarifer* Jałoszyński, *V.gansuanus* Jałoszyński, *V.shaanxiana* Jałoszyński, and *V.dabashana* Jałoszyński, recorded in China have morphological characters similar to those of the Japanese species groups but do not perfectly align with their characteristics. Therefore, it is difficult to assign these species to any specific group (Jałoszyński 2009, 2012, 2013, 2024).

The genus *Veraphis* was first recorded from the Korean Peninsula in 2005, with the description of *V.engelmarkikoreanus* Jałoszyński & Hoshina, 2005 from Pyongyang, North Korea. Since then, no additional species of *Veraphis* have been documented from the Korean Peninsula. This study reports two new species of *Veraphis* from South Korea, marking the first record in approximately 20 years. The present study provides images of the habitus, aedeagus, and a distribution map.

## ﻿Material and methods

A total of 14 dry and alcohol-preserved specimens were examined in this study. Male specimens were relaxed in warm water before being mounted on sticky tabs for imaging. Subsequently, the specimens were point-mounted and preserved as dry specimens. Observations were conducted using a Leica S8APO. Images of the habitus, diagnostic characters, and aedeagus were produced using a Sony ILCE-7RM3 mirrorless camera with a Mitutoyo M Plan Apo 20X objective lens. The aedeagus was imaged after the internal organs were removed using Proteinase K. The images were stacked using Helicon Focus 8, and line drawings were created with Adobe Illustrator 2024. Morphological terminology followed Jałoszyński (2014b) and Jałoszyński and Hoshina (2005). Distribution maps were generated using SimpleMappr (Shorthouse 2010). The figure plates used in this study were edited and produced using Adobe Photoshop 2024. Data labels of holotypes were transcribed verbatim, while those of paratypes were standardized for consistency. Body length was measured from the anterior margin of the head to the posterior margin of the elytra.

All examined specimens were deposited in the following collections.

**CBNUIC**Chungbuk National University Insect Collection, Cheongju, South Korea;

**NIBR**National Institute of Biological Resources, Incheon, South Korea.

## ﻿Taxonomy

### ﻿Genus Veraphis

Taxon classificationAnimaliaColeopteraStaphylinidae

Casey, 1897

BC05165F-F3C4-5F5A-8764-6872BED82206

Veraphis Casey, 1897: 509. Type species: Eutheiaimpressa LeConte, 1879 (designated by Franz in Newton and Franz 1998).

#### Diagnosis.

**Figure 1.**
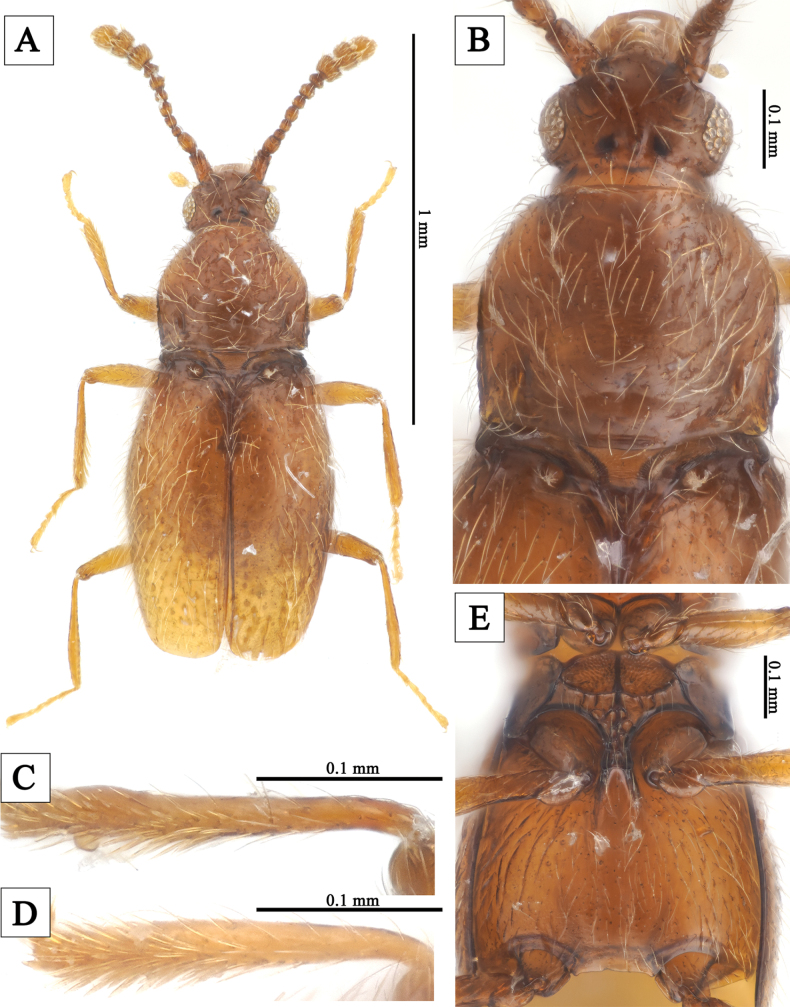
*Veraphisodaesanensis* sp. nov. **A** male habitus, dorsal view **B** male head and pronotum, dorsal view **C** male protibia **D** female protibia **E** meso-, metaventrite.

**Figure 2.**
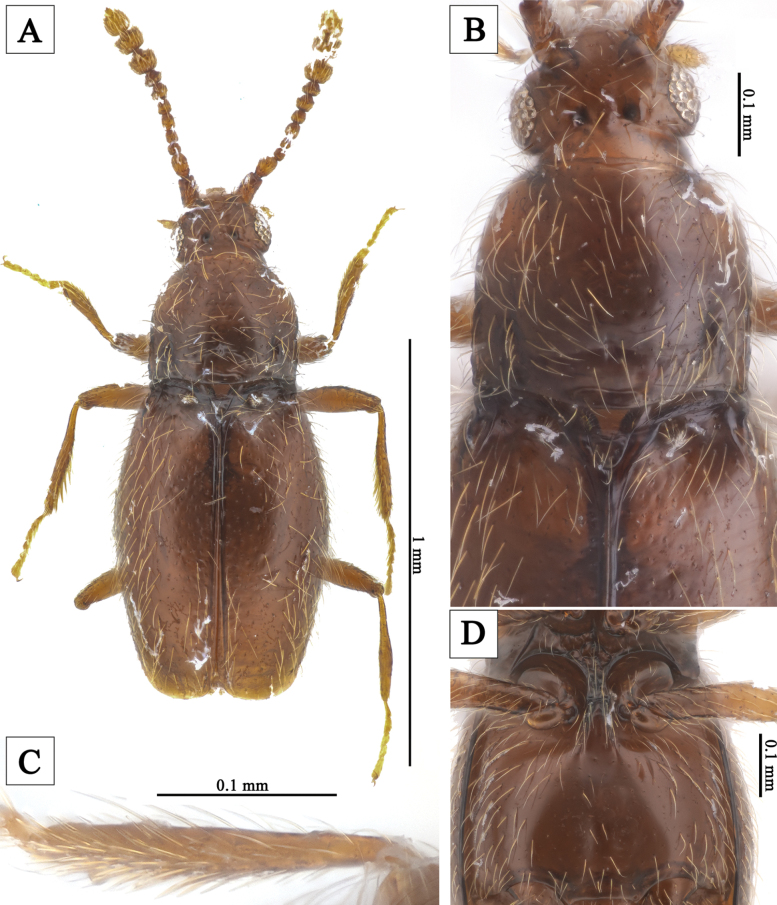
*Veraphismyeonggiensis* sp. nov. **A** male habitus, dorsal view **B** male head and pronotum, dorsal view **C** male protibia **D** meso-, metaventrite.

This genus is distinguished from other genera of Scydmaeninae by the following combination characteristics: body flattened and elongated (Figs 1A, 2A); procoxal cavities broadly open (Fig. 1E); prosternum with a narrow intercoxal carina (Fig. 1E); each elytron with a setose basal fovea (Figs 1B, 2B). For more detailed diagnostic characters and phylogenetic information on this genus, refer to Jałoszyński (2014b).

#### Distribution.

Northern Hemisphere (China, Japan, Korea, USA, Finland, Sweden, Mongolia, Russia (Far East, East Siberia)).

#### Remarks.

According to Jałoszyński 2024, this genus is primarily found in leaf litter and soil, and all species discovered in China inhabit alpine regions at altitudes above 2000 m. Additionally, species found in Japan are also thought to be distributed in cooler climates. These species are very similar in external morphology, making examination of the aedeagus crucial for species identification. In some species, modifications of the male legs are present, which can be useful for distinguishing species.

### ﻿ Veraphis odaesanensis sp. nov.

Taxon classificationAnimaliaColeopteraStaphylinidae

375C7829-7D08-5017-B1EA-315EB6007720

https://zoobank.org/99E3D6D4-1540-4CA5-81F9-FA16CD4DC18F

Figs 1 , 3A–D , 4

#### Type material designated.

***Holotype*** • ♂ NIBR: “**Korea**: Gangwon Prov. Mt. Odae, Dongsan-ri, Jinbu-myeon, Pyeongchang-gun, 17.VIII.2022, 37°47'14.2"N, 128°33'56.2"E, 897 m, sifting leaf & soil litter, J.-W. Seo, J.-I. Shin” ***Paratypes* Korea**: Gangwon Prov. • 1♂1♀ (1♂ genitalia dissected; CBNUIC), Pyeongchang-gun, Jinbu-myeon, Dongsan-ri, 21.IX–02.XI.2022, 37°47'14.2"N, 128°33'56.2"E, 897 m, F.I.T, J.-W. Kang, J-I. Shin • 1♀ (CBNUIC), 02.XI.2022, 37°47'14.2"N, 128°33'56.2"E, 897 m, F.I.T, J.-W. Kang, J-I. Shin • 1♀ (CBNUIC), 17.VIII.2022, 37°47'14.2"N, 128°33'56.2"E, 897 m, sifting soil & leaf litter, J.-W. Seo, J-I. Shin • 8♀♀ (7♀♀ 95% EtOH in tube; CBNUIC), Odaesan-ro 1211-14, Mt. Odae, Sangwonsa, 17.VIII–21.IX.2022, 37°47'14.2"N, 128°33'56.2"E, 897 m, F.I.T, J-I. Shin U.-J. Hwang.

#### Diagnosis.

**Figure 3.**
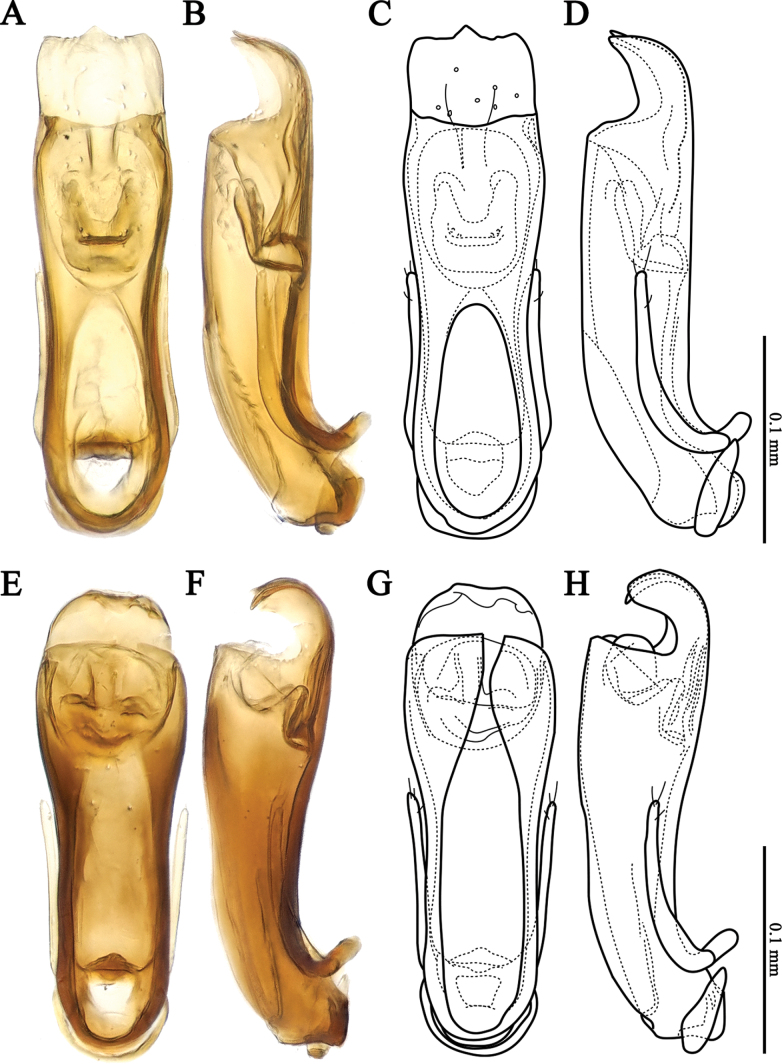
Aedeagus of *Veraphisodaesanensis* sp. nov. (**A–D**) and *V.myeonggiensis* sp. nov. (**E–H**) **A, C, E, G** ventral view **B, D, F, H** lateral view.

Vertex with two shallow longitudinal grooves extending from posterior margin to posterior 1/2; area between grooves relatively flattened and impressed (Fig. 1B). Protibiae of male with small subapical pin-like projection (Fig. 1D). Metaventrite with shallow longitudinal impression (Fig. 1E). Aedeagus (Fig. 3A–D) strongly elongated and symmetrical, length 0.24 mm. In ventral view, lateral margins of median lobe somewhat parallel, middle of apex weakly protruding, with small shallow punctures in subapical region in ventral view; in lateral view, slightly curved near base and subapical region with strong ventral indentation. Endophallus symmetrical, U-shaped structured. Parameres slender, reaching middle of median lobe; each paramere with two short apical setae and one subapical seta.

#### Male description.

Body length 1.27–1.32 mm; reddish-brown, appendages lighter; flattened and elongated; surface covered yellow hairs (Fig. 1A). Head wider than long, widest across eyes; punctures of surface inconspicuous; hairs short and sparse (Fig. 1B). Temples nearly 1/4 length of eyes, with shorter hairs than those on surface (Fig. 1B). Vertex with small pits on medioposterior margin; two shallow longitudinal grooves extending from posterior margin to posterior 1/2 present; area between grooves relatively flattened and impressed (Fig. 1B). Antennae with distinct distal three-segmented club; antennomere 1 strongly elongate, 2 elongate but weaker than 1, 3 slightly wider than long, 4–6 as long as wide, 5 slightly larger than 4, 6 slightly smaller than 5, 7 subpentagonal, 8 distinctly wider than long, 9–11 forming club (Fig. 1A). Pronotum distinctly wider than head, as long as wide, widest near middle; anterior margin somewhat rounded, lateral margins strongly rounded in anterior 1/3, somewhat parallel in posterior 1/3, posterior angles somewhat right-angled, posterior margin weakly sinuate; pronotal base with shallow median pits and transverse impression, lateral pits distinct; punctures of surface inconspicuous; hairs short and sparse (Fig. 1B). Elytra slightly wider than pronotum, distinctly longer than wide, widest near middle; lateral margins and posterior margin relatively rounded; punctures of surface inconspicuous; hairs short and sparse; each elytron with distinct humeral denticle (Fig. 1A, B). Hind wings well-developed. Metaventrite with shallow longitudinal impression (Fig. 1E). Legs moderately long and slender. Protibiae with small subapical pin-like projection (Fig. 1C). Aedeagus (Fig. 3A–D) strongly elongated and symmetrical, length 0.24 mm. In ventral view, lateral margins of median lobe somewhat parallel, middle of apex weakly protruding, with small shallow punctures in subapical region in ventral view; in lateral view, slightly curved near base and subapical region with strong ventral indentation. Endophallus symmetrical, U-shaped. Parameres slender, reaching middle of median lobe; each paramere with two short apical setae and one subapical seta.

#### Sexual dimorphism.

Protibiae without subapical pin-like projection (Fig. 1D).

#### Distribution.

**Figure 4.**
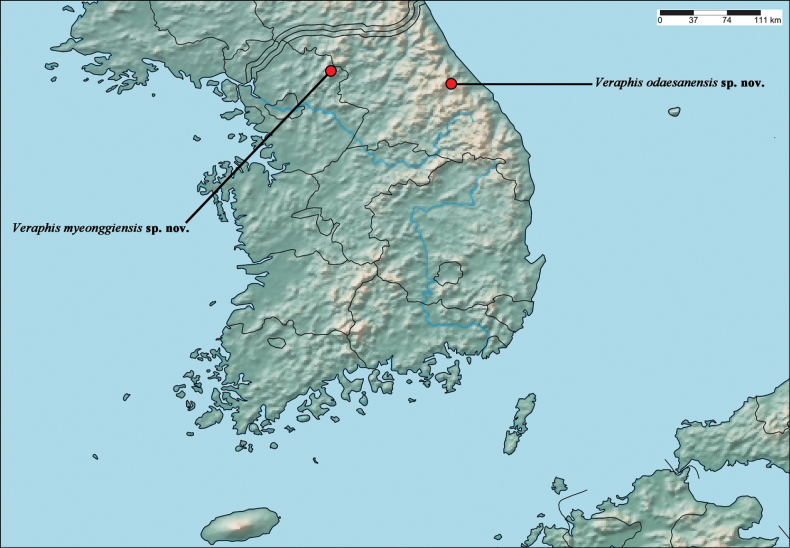
Distribution map.

South Korea (Fig. 4).

#### Etymology.

The specific epithet is an adjective derived from the type locality ‘Mt. Odaeʼ.

#### Habitat.

This species was collected from relatively high-altitude mountains in South Korea, at elevations above 800 m. It was frequently captured using flight intercept traps, indicating its ability to fly with well-developed wings. Additionally, it was also collected from soil and leaf litter.

#### Remarks.

*Veraphisodaesanensis* can be classified within the *japonicus* species group based on the characteristics of the male leg, antennae, and aedeagus (Jałoszyński and Hoshina 2005). This species shows clear differences in the aedeagus from the Japanese species. In China, it shares a similar aedeagus with *V.assingi* Jałoszyński, but the apex of the median lobe is more pointed, and there are significant differences in the morphology of the endophallus and the median lobe in lateral view.

### ﻿ Veraphis myeonggiensis sp. nov.

Taxon classificationAnimaliaColeopteraStaphylinidae

34D920B8-BC1E-57EB-8786-C07B8F6379D4

https://zoobank.org/352AF5C9-41F3-43D4-8835-2A12E1807574

Figs 2 , 3E–H , 4

#### Type material designated.

***Holotype*** • ♂ NIBR: “**Korea**: Gyeonggi Prov. Mt. Myeonggi, 520, Nonnamgi-gil, Buk-myeon, Gapyeong-gun, 5.X.2023, 37°58'12.2"N, 127°24'18.7"E, 402 m, sifting leaf litter & soil, J.-W. Kang, J.-I. Shin”.

#### Diagnosis.

Vertex with two shallow longitudinal grooves extending from posterior margin to posterior 1/2; area between grooves relatively flattened and impressed (Fig. 2B). Protibiae of male with small subapical pin-like projection (Fig. 2C). Metaventrite with shallow longitudinal impression (Fig. 2D). Aedeagus (Fig. 3E–H) strongly elongated and symmetrical, length 0.23 mm. In ventral view, median lobe gradually widening from base to apical 1/4, widest at apical 1/4, then slightly narrowing; sides from base to middle somewhat parallel; apex rounded; in lateral view, slightly curved near base and subapical region with strong ventral indentation. Endophallus symmetrical, structure complex. Parameres slender, reaching middle of median lobe; each paramere with two short apical setae and one subapical seta.

#### Male description.

Body length 1.14 mm; reddish-brown, appendages lighter; flattened and elongated; surface covered yellow hairs (Fig. 2A). Head wider than long, widest across eyes; punctures of surface inconspicuous; hairs short and sparse (Fig. 2B). Temples nearly 1/4 length of eyes (Fig. 2B). Vertex with small pits on medioposterior margin; two shallow longitudinal grooves extending from posterior margin to posterior 1/2 present; area between grooves relatively flattened and impressed (Fig. 2B). Antennae with distinct distal three-segmented club; antennomere 1 strongly elongate, 2 elongate but less so than 1, 3 slightly wider than long, 4–6 as long as wide, 5 slightly larger than 4, 6 slightly smaller than 5, 7 subpentagonal, 8 distinctly wider than long, 9–11 forming a club (Fig. 2A). Pronotum distinctly wider than head, as long as wide, widest near middle; anterior margin somewhat rounded, lateral margins strongly rounded in anterior 1/3, somewhat parallel in posterior 1/3, posterior angles somewhat right-angled, posterior margin weakly sinuate; pronotal base with shallow median pits and transverse impression, lateral pits distinct; punctures of surface inconspicuous; hairs short and sparse (Fig. 2B). Elytra slightly wider than pronotum, distinctly longer than wide, widest near middle; lateral margins and posterior margin relatively rounded; punctures of surface inconspicuous; hairs short and sparse; each elytron with distinct humeral denticle (Fig. 2A, B). Hind wings well-developed. Metaventrite with shallow longitudinal impression (Fig. 2D). Legs moderately long and slender. Protibiae with small subapical pin-like projection (Fig. 2C). Aedeagus (Fig. 3E–H) strongly elongated and symmetrical, length 0.23 mm. In ventral view, median lobe gradually widening from base to apical 1/4, widest at apical 1/4, then slightly narrowing; sides from base to middle somewhat parallel; apex rounded; in lateral view, slightly curved near base and subapical region with strong ventral indentation. Endophallus symmetrical, complex structured. Parameres slender, reaching middle of median lobe; each paramere with two short apical setae and one subapical seta.

#### Sexual dimorphism.

Unknown.

#### Distribution.

South Korea (Fig. 4).

#### Etymology.

The specific epithet is an adjective derived from the type locality ‘Mt. Myeonggiʼ.

#### Habitat.

This species was collected from soil and leaf litter in mixed forest at relatively low altitudes, unlike other previously known species.

#### Remarks.

*Veraphismyeonggiensis* can be classified within the *japonicus* species group based on the characteristics of the male leg, antennae, and aedeagus (Jałoszyński and Hoshina 2005). The aedeagus of this species is similar to that of *V.tottoriensis* Jałoszyński & Hoshina from Japan. However, the apex of the median lobe is more strongly curved ventrally, and the central structure of the endophallus is absent. Additionally, the parameres are slightly shorter. It also shares a similar aedeagus with *V.modestus* Jałoszyński but differs in the apex of the median lobe and the structure of the endophallus. Moreover, the overall morphology in lateral view is clearly distinct. Also, this species is externally very similar to *E.odaesanensis*, but it is smaller in size and clearly differed in the aedeagus.

## Supplementary Material

XML Treatment for Veraphis

XML Treatment for Veraphis odaesanensis

XML Treatment for Veraphis myeonggiensis

## ﻿Additional information

### Author ORCIDs

Ui-Joung Byeon https://orcid.org/0009-0000-0512-2312

Jong-Seok Park https://orcid.org/0000-0003-2063-2534

### Data availability

All of the data that support the findings of this study are available in the main text.
